# A fast Newton–Raphson based iterative algorithm for large scale optimal contribution selection

**DOI:** 10.1186/s12711-016-0249-2

**Published:** 2016-09-20

**Authors:** Binyam S. Dagnachew, Theo H. E. Meuwissen

**Affiliations:** Department of Animal and Aquacultural Sciences, Norwegian University of Life Sciences, P.O. Box 5003, 1432 Ås, Norway

## Abstract

**Background:**

The management of genetic variation in a breeding scheme relies very much on the control of the average relationship between selected parents. Optimum contribution selection is a method that seeks the optimum way to select for genetic improvement while controlling the rate of inbreeding.

**Methods:**

A novel iterative algorithm, Gencont2, for calculating optimum genetic contributions was developed. It was validated by comparing it with a previous program, Gencont, on three datasets that were obtained from practical breeding programs in three species (cattle, pig and sheep). The number of selection candidates was 2929, 3907 and 6875 for the pig, cattle and sheep datasets, respectively.

**Results:**

In most cases, both algorithms selected the same candidates and led to very similar results with respect to genetic gain for the cattle and pig datasets. In cases, where the number of animals to select varied, the contributions of the additional selected candidates ranged from 0.006 to 0.08 %. The correlations between assigned contributions were very close to 1 in all cases; however, the iterative algorithm decreased the computation time considerably by 90 to 93 % (13 to 22 times faster) compared to Gencont. For the sheep dataset, only results from the iterative algorithm are reported because Gencont could not handle a large number of selection candidates.

**Conclusions:**

Thus, the new iterative algorithm provides an interesting alternative for the practical implementation of optimal contribution selection on a large scale in order to manage inbreeding and increase the sustainability of animal breeding programs.

## Background

Advancements in genetic evaluation methods such as the use of best linear unbiased prediction (BLUP) have substantially increased response to selection in modern animal breeding programs. Selection programs are usually designed to optimize genetic gain with no or an implicit limitation on the rate of inbreeding (for example [1]). However, although inbreeding cannot be avoided in closed selection programs, rates of inbreeding need to be controlled to prevent long-term negative effects of selection [2–4].

Although the main goal in breeding programs is to maximize genetic gain, management of inbreeding is vital for the sustainability of breeding schemes. The optimal balance between rate of inbreeding ($\Delta F$) and genetic gain ($\Delta G$) is a core problem in practical animal breeding. In the late 1990s, a dynamic selection method, known as optimum contribution (OC) selection, was proposed to deal with this problem of optimization [5–7]. OC selection attempts to maximize genetic response for a given rate of inbreeding (i.e. as influenced by relationships among selection candidates) by considering the genetic contribution of candidates and using the numerator relationship matrix (${\mathbf{A}}$).

Simulation results showed that OC selection could achieve up to 60 % more genetic gain compared with truncation selection at the same rate of inbreeding [5, 6]. The potential application of the OC selection method in practical breeding programs has also been studied, for instance, in dairy cattle [8, 9], in British sheep and beef cattle [10] and in salmon [11]. These studies reported greater genetic gain under OC selection at the same inbreeding rate compared to traditional truncation selection.

Several algorithms have been developed for the optimization of genetic gain and rate of inbreeding, such as evolutionary algorithms [12, 13], genetic algorithms [14], and semi-definite programming [7]. Meuwissen [6, 15] presented an optimization algorithm using Lagrangian multipliers, i.e. Gencont, to calculate optimized genetic contributions of selection candidates that were constrained on a predefined rate of inbreeding ($\Delta F$). The algorithm requires inversion of the relationship matrix of the selection candidates, which needs to be re-calculated several times since some candidates prove unfit for selection (i.e. candidates with poor breeding values). However, there are challenges for implementing this in practical breeding schemes, which have large numbers of selection candidates, due to computational limitations of repeated calculation of the inverses of large matrices.

Hinrichs et al. [11] developed an alternative algorithm, based on Meuwissen’s approach [6], called OCSelect. The algorithm avoids the actual setting up of the inverse of a relationship matrix between selection candidates by partitioning the matrix into a diagonal matrix and an inverse of a relationship matrix between parents of the candidates [11]. The main assumption is that the latter matrix has a relatively small size and it is easier to find the inverse. This might be the case in aquaculture breeding schemes, where the OCSelect algorithm is developed and tested on; however, it might not be the case in dairy cattle and sheep breeding schemes, where large numbers of parents are involved.

An alternative approach considered here is to obtain optimum contributions iteratively without calculating the inverse of the relationship matrix. Therefore, this paper presents an iterative algorithm, referred as to Gencont2, for the calculation of optimized genetic contributions.

## Methods

### Theory

The main goal of selection schemes is to maximize the genetic level of the next generation. Let $\Delta G$ be the genetic level of the next generation, which can be expressed as:

$$\Delta G = {\mathbf{c}}^{{T}} {\mathbf{EBV}},$$

where ${\mathbf{EBV}}$ is a vector of estimated breeding values of selected parents and ${\mathbf{c}}$ is a vector of genetic contributions of the selected parents to the next generation. The problem is to find the optimum contribution, ${\mathbf{c}}$. Therefore, $c_{i} \ge 0$ for a candidate *i*, and with the total contribution summing to 1 (i.e. $\sum\nolimits_{i = 1}^{n} {c_{i} = 1}$). In diploid species, each sex contributes half of the genes to the gene pool (i.e. $\sum {c_{i} } = 0.5$ where the sum is over all individuals of a sex). Then, the restriction on contribution per sex is:

1 $${\mathbf{c}}^{{{T}}} {\mathbf{Q}} = {\mathbf{r}},$$

where ${\mathbf{Q}}$ is a known incidence matrix for the sex of the candidates, and **r** is a vector of 0.5 s of length 2. Control of inbreeding is achieved by constraining the group co-ancestry of selected candidates. For a set of genetic contributions of selected candidates, the constraint on the group co-ancestry is:

2 $$K = \left( {\frac{1}{2}} \right)\left( {{\mathbf{c}}^{T} {\mathbf{Ac}}} \right),$$

where ${\mathbf{A}}$ is the additive relationship matrix of the selection candidates and $K$ is the value of the constraint. The co-ancestry constraint, $K$, was set to follow a path at a rate of inbreeding $\Delta F$, i.e. $K = C_{p} +\Delta F(1 - C_{p} )$, where $C_{p}$ is the average co-ancestry of the current population or the co-ancestor constraint $K$ used in the previous generation, and $\Delta F$ is the targeted rate of inbreeding.

The optimal $c$ that maximizes $\Delta G$ under the above constraints [i.e. Eqs. (1), (2)] is then obtained by maximizing the following Lagrangian multipliers objective function:

$$H = {\mathbf{c}}^{{{T}}} {\mathbf{EBV}} - \lambda_{0} \left( {2K - {\mathbf{c}}^{{{T}}} {\mathbf{Ac}}} \right) - {\varvec{\uplambda}}\left( {{\mathbf{c}}^{{{T}}} {\mathbf{Q}} - {\mathbf{r}}} \right),$$

where $\lambda_{0}$ and ${\varvec{\uplambda}}$ are the LaGrangian multipliers (${\varvec{\uplambda}}$ is a vector of length 2). Maximizing the objective function for ${\mathbf{c}}$ yields:

3 $${\mathbf{Ac}} = \left( {{\mathbf{EBV}} - {\mathbf{Q}}{\varvec{\uplambda}}} \right)/2\lambda_{0} ,$$

From the constraint in Eq. (2) follows an equation for $\lambda_{0}$:

4 $$\lambda_{0}^{2} = \frac{{{\mathbf{EBV}}^{T} \left( {{\mathbf{A}}^{{ - {\mathbf{1}}}} - {\mathbf{A}}^{{ - {\mathbf{1}}}} {\mathbf{Q}}\left( {{\mathbf{Q}}^{{T}} {\mathbf{A}}^{{ - {\mathbf{1}}}} {\mathbf{Q}}} \right)^{ - 1} {\mathbf{Q}}^{{T}} {\mathbf{A}}^{{ - {\mathbf{1}}}} } \right){\mathbf{EBV}}}}{{8K - {\mathbf{1}}^{T} \left( {{\mathbf{Q}}^{{T}} {\mathbf{A}}^{{ - {\mathbf{1}}}} {\mathbf{Q}}} \right)^{ - 1} {\mathbf{1}}}},$$

where ${\mathbf{1}}$ is a vector of ones. For a more detailed derivation of Eqs. (3) and (4) see [6]. The procedure to find optimal solutions iteratively is described in the following five steps.

Step 1: Calculate the starting values for $\lambda_{0}$ by solving Eq. (4) (assuming ${\mathbf{A}}^{{ - {\mathbf{1}}}} = {\mathbf{I}}$) and initiate some starting values for ${\varvec{\uplambda}}$ (for instance zeros). Then, calculate optimal contributions, ${\mathbf{c}}$, by solving Eq. (3), i.e. ${\mathbf{Ac}} = \left( {{\mathbf{EBV}} - {\mathbf{Q}}\varvec{\uplambda}} \right)/2\lambda_{0}$, using the Gauss–Seidel method.

Step 2: Update ${\varvec{\uplambda}}$ by taking the gradient of Eq. (3) with respect to ${\varvec{\uplambda}}$ and rearranging:

$${\mathbf{Q}}^{{T}} {\varvec{\partial }}{\mathbf{c}} = - {\mathbf{Q}}^{{T}} {\mathbf{A}}^{{ - {\mathbf{1}}}} {\mathbf{Q}}{\varvec{\partial \uplambda }}/2\lambda_{0} .$$

Let $\Delta {\mathbf{r}}$ be the derivation from the constraint ${\mathbf{Q}}^{T} {\mathbf{c}} = {\mathbf{r}}$ [Eq. (1)], $\Delta {\mathbf{r}} = {\mathbf{r}} - {\mathbf{Q}}^{T} {\mathbf{c}}$. We want to change ${\mathbf{c}}$ by ${\varvec{\partial }}{\mathbf{c}}$ such that $\Delta {\mathbf{r}} - {\mathbf{Q}}^{T} {\varvec{\partial }}{\mathbf{c}} = 0$ (i.e. $\Delta {\mathbf{r}} = {\mathbf{Q}}^{T} {\varvec{\partial }}{\mathbf{c}}$). Assuming ${\mathbf{A}}^{{ - {\mathbf{1}}}} = {\mathbf{I}}$, and rearranging:

$$\partial {\varvec{\uplambda}} = - 2*({\mathbf{Q}}^{T} {\mathbf{Q}})^{ - 1} *\Delta {\mathbf{r}}*\lambda_{0} ,$$

and $${\varvec{\uplambda}}_{{{\mathbf{new}}}} = {\varvec{\uplambda}} + {\varvec{\partial }}{\varvec{\uplambda}}$$,

Step 3: Update $\lambda_{0}$, by taking the gradient of Eq. (3) with respect to $\lambda_{0}$ and rearranging:

$${\varvec{\partial }}{\mathbf{c}} = - \frac{{{\mathbf{A}}^{{ - {\mathbf{1}}}} \left( {{\mathbf{EBV}} - {\mathbf{Q\lambda }}} \right)\partial \lambda_{0} }}{{2\lambda_{0}^{2} }}.$$

Let ${{\Delta }}K$ be the derivation from the constraint $K = \left( {1/2} \right)\left( {{\mathbf{c}}^{{{T}}} {\mathbf{Ac}}} \right)$ [Eq. (2)], $\Delta K = 2K - {\mathbf{c}}^{T} {\mathbf{Ac}}$. We want to change ${\mathbf{c}}$ by ${\varvec{\partial }}{\mathbf{c}}$ such that $\Delta K - 2\left( {{\mathbf{c}}^{T} {\mathbf{A}}} \right){\varvec{\partial }}{\mathbf{c}} = 0$ (i.e. $\Delta K = 2\left( {{\mathbf{c}}^{{{T}}} {\mathbf{A}}} \right){\varvec{\partial }}{\mathbf{c}}$). Thus, rearranging:

$$\partial \lambda_{0} = - \frac{{ {{\Delta }}K\lambda_{0}^{2} }}{{{\mathbf{c}}^{{{T}}} \left( {{\mathbf{EBV}} - {\mathbf{Q}}{\varvec{\uplambda}}} \right)}},$$

and $$\lambda_{{0}_{new}} = \lambda_{0} + \partial \lambda_{0}$$,

Step 4: Re-calculate optimal contributions using the updated values for $\lambda_{{0_{new} }}$ and ${\varvec{\uplambda}}_{{{\mathbf{new}}}}$ by solving Eq. (3) for ${\mathbf{c}}$, i.e. by solving ${\mathbf{Ac}} = \left( {{\mathbf{EBV}} - {\mathbf{Q}}{\varvec{\uplambda}}} \right)/2\lambda_{0}$ using the Gauss–Seidel method.

Step 5: Check for convergence and that the solutions are valid. The convergence indicator was the sum of the squares of the difference between consecutive iterative solutions for ${\mathbf{c}}$:

$$sse_{n} = \frac{{\left( {{\mathbf{c}}_{{{\mathbf{n}} - {\mathbf{1}}}} - {\mathbf{c}}_{{\mathbf{n}}} } \right)^{T} \left( {{\mathbf{c}}_{{{\mathbf{n}} - {\mathbf{1}}}} - {\mathbf{c}}_{{\mathbf{n}}} } \right)}}{{({\mathbf{c}}_{{\mathbf{n}}} )^{T} ({\mathbf{c}}_{{\mathbf{n}}} )}},$$

where the subscript $n$ represent the iteration number. Further steps to check that solutions are valid and the constraint is met are done by monitoring $\Delta {\mathbf{r}}$ and ${{\Delta }}K$, respectively. Convergence was monitored after each round of iteration. If it does not converged ($sse_{n} > 10^{ - 6}$ or $\Delta {\mathbf{r}} > 10^{ - 4}$ or $\Delta K > 10^{ - 4}$), the algorithm will return back to step 2.

Due to the lack of a constraint that makes sure that all contributions are valid (i.e. $c_{i} \ge 0$), some of the solutions could be negative for some candidates with lower ${\text{EBV}}$. Meuwissen [6] solved this by fixing the contributions of these candidates to 0 and eliminating the animals from the optimization process and repeating the process until no solutions are negative. This might lead to suboptimal solutions under some circumstances [7]. In the iterative algorithm, while solving Eq. (3) using the Gauss–Seidel method, solutions are constrained to be valid (i.e. solutions are either zero or positive). This approach is similar to solutions that are subjected to a constraint $c_{i} \ge 0$. Unlike Gencont, this avoids the need to remove animals from the optimization process to obtain valid solutions. However, for computational reasons, animals with zero contributions were removed after 500 iterations for the first time and after every 100th iteration until convergence (note: removal of individuals with 0 contribution is done only for computational purposes and one can implement the algorithm without this step).

In principle, it is possible that the contribution from a single candidate is very high. However, due to biological limits to reproductive capacity or management policies, the maximum contribution per candidate may be restricted to a value less than 0.5. Let ${\mathbf{Cmax}}$ be a vector containing the maximum contribution for each candidate, where the maximum contribution $Cmax_{i}$ may vary across candidates and $Cmax_{i} = 0.5$ for candidates with no maximum restriction. A restriction on the minimum contribution (${\mathbf{Cmin}}$) for each candidate can also be set where a selected animal gets contribution ≥$Cmin_{i}$ (i.e. contribution is either zero or ≥$Cmin_{i}$). A predefined number of selected dams, $Ndams$ is obtained by setting $Cmin = Cmax = 0.5/Ndams$. An extension of the iterative algorithm to accommodate $Cmin\;{\text{and}}\;Cmax$ is described in the Appendix.

The presented algorithm considers only discrete generations. It is extended to handle overlapping generations following the method presented in [16]. This iterative algorithm, referred to as Gencont2, was programmed in FORTRAN95 language. The program is available upon request.

### Description of datasets

The performance of the program was tested on real datasets obtained from three practical breeding programs and results were compared with the original algorithm, Gencont [15]. The datasets were obtained from Geno (a cattle breeding organization for the Norwegian Red breed), Norsvin (a Norwegian swine breeding organization) and NSG (the Norwegian association of sheep and goat farmers). For the *Cattle dataset*, the number of selection candidates was 3907, the pedigree file contained 23,224 animals and the EBV were estimated by BLUP. For the *Pig dataset*, the number of selection candidates was 2929, the pedigree file contained 11,945 animals and the EBV were based on index scores. For the *Sheep dataset*, the number of selection candidates was 6875, the pedigree file contained 82,225 animals and the EBV were based on index scores (Table 1). In these datasets, all selection candidates were males.

**Table 1:** Description of datasets

Dataset	Number of selection candidates	Pedigree size	EBV			Average relationship^a^	Average inbreeding^b^
			Min	Mean	Max		
Cattle	3907	23,224	0.595	1.118	1.569	0.04234	0.01709
Pig	2929	11,945	88.50	111.8	134.0	0.1928	0.09269
Sheep	6875	82,225	63.00	123.6	156.0	0.14595	0.02771

^a^Average relationship between selection candidates

^b^Average inbreeding of selection candidates

Initially, the datasets were analyzed without any restriction on the reproductive capacities of the selection candidates. However, subsequent analyses were carried out with restrictions $Cmax$ and $Cmin$. Restriction on the minimum contribution ($Cmin$) implies that, for the $ith$ animal to be selected, it had to contribute at least this amount (i.e. $Cmin_{i}$). However, restriction on the maximum contribution ($Cmax$) implies that an animal should not contribute more than the stated amount (i.e. $Cmax_{i}$). $Cmin$ was set at 0.5 or 0.25 % and $Cmax$ was set at 1, 2, 3, 4, and 5 %. The datasets were analyzed with different combinations of $Cmin$ and $Cmax$. A scenario where $Cmin = Cmax$ was also tested by specifying predefined numbers of candidates to select. For the cattle dataset, 120 bulls were defined to be selected, which gave $Cmin = Cmax = 0.883\,\%$. For the pig datasets 110 boars were defined to be selected, which gave $Cmin = Cmax = 0.9\,\%$. Attributes of the OC selection were examined including the optimal number of selected animals, the genetic merit of the selected parents, achievement of the imposed constraints, and computer time. All computations were done on the Abel clusters that are owned by the University of Oslo and the Norwegian metacentre for high performance computing.

## Results and discussion

**Fig. 1:**
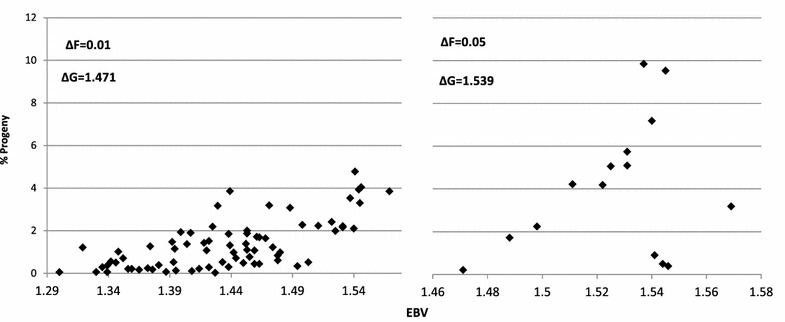
Association between EBV and optimized genetic contribution for the selected candidates in the cattle dataset by applying two levels of constraints on rate of inbreeding $(\Delta F)$). $\Delta G$ = genetic gain

This paper presents a novel iterative algorithm, Gencont2, for calculating optimized genetic contributions with a predefined rate of inbreeding. Figure 1 shows the association between EBV and optimized genetic contributions of selected bulls for the cattle dataset with a targeted rate of inbreeding of 0.01 (left) and 0.05 (right). The algorithm was successful in constraining $\Delta F$ to the predefined levels. The number of selected bulls (with nonzero contributions) increased when more severe constraints were placed on $\Delta F$ (Fig. 1). For example, the number of animals selected increased from 15 to 75 as the allowed rate of inbreeding decreased from 0.05 to 0.01. Consequently, the expected genetic gain also decreased as the constraint became more stringent (Fig. 1). These results are expected because as more severe restrictions are placed on future inbreeding, contributions from superior animals will decrease and more animals are selected in order to achieve the average relationship constraint. For instance, the maximum percentage of progeny per individual was well below 5 % when $\Delta F$ was 0.01 compared with 10 % when $\Delta F$ was 0.05 (Fig. 1). Similar results were reported in other OC selection studies [8, 11].

Results of the current algorithm were compared with Gencont [15] on the basis of the optimal number of selected animals, genetic gain, average relationship of the parents, and computer time. Analyses of the cattle dataset using both algorithms at different rates of inbreeding are summarized in Table 2. Both algorithms suggested that equal optimal numbers of candidates at 0.05 and 0.01 rates of inbreeding were selected and gave similar results with respect to genetic gain. The candidates that were selected by the two algorithms were the same and the assigned contributions had correlations very close to 1 (Table 2). For inbreeding rates of 0.005 and 0.001, the iterative algorithm selected fewer candidates and yielded a slightly higher genetic gain. However, the additional selected candidates had very low assigned contributions that ranged from 0.006 to 0.03 % and both algorithms successfully met the constraints. The iterative algorithm considerably reduced the computer time by around 92 % compared to Gencont (Table 2).

**Table 2:** Analysis of *Cattle dataset* using Gencont2 and Gencont

$\Delta F$	$\Delta G$ ^a^	Ave_relationship^b^	Number of selected candidates^a^	Time^c^	R^d^
0.05	1.539	0.14057	15	8.1	0.999
0.01	1.471	0.06227	75	7.9	0.999
0.005	1.449 (1.448)	0.05249	104 (106)	7.9	0.998
0.001	1.424 (1.423)	0.04465	127 (128)	7.9	0.996

Different levels of rate of inbreeding with respect to genetic gain ($\Delta G$), number of selected individuals relative necessary computation time and correlation between assigned contributions

^a^If there was difference between the two algorithms, the result obtained with Gencont is shown in parentheses

^b^Average relationship between selected candidates

^c^Amount of computation time necessary for Gencont2 expressed as the fraction of the time necessary for Gencont (in %)

^d^Correlation between assigned contributions

The optimal number of boars selected, expected genetic gain and relative computer times using both algorithms at different inbreeding rates for the pig dataset are in Table 3. The optimal number of selected boars ranged from 28 to 103 for different levels of constraints on inbreeding rate. Both algorithms gave very similar results with respect to the optimal number of boars to select and genetic gains when the predefined inbreeding rates were 0.05 and 0.001 (Table 3). The iterative algorithm suggests that fewer boars would be selected when constraints on inbreeding rate were 0.01 and 0.005. The different selected candidates have contributions that ranged from 0.02 to 0.08 % and the correlations between assigned contributions by the two algorithms were higher than 0.98 (Table 3). For all these analyses, both algorithms satisfied the imposed constraints. The iterative algorithm used only from 6.5 to 9.3 % of the computer time to obtain optimal solutions compared to Gencont (Table 3).

**Table 3:** Analysis of the *Pig dataset* using Gencont2 and Gencont

$\Delta F$	$\Delta G$ ^a^	Ave_relationship^b^	Number of selected candidates^a^	Time^c^	R^d^
0.05	129.55	0.28481	28	6.8	0.999
0.01	125.43 (125.39)	0.21259	73 (77)	6.5	0.986
0.005	124.37 (124.42)	0.20356	84 (90)	8.2	0.986
0.001	123.40 (123.44)	0.19634	103	9.3	0.990

Different levels of rate of inbreeding with respect to genetic gain ($\Delta G$), number of selected individuals relative necessary computation time and correlation between assigned contributions

^a^If there was a difference between the two algorithms, the result obtained with Gencont is shown in parentheses

^b^Average relationship between selected candidates

^c^Amount of computation time necessary for Gencont2 expressed as the fraction of the time necessary for Gencont (in  %)

^d^Correlation between assigned contributions

Both methods use the same approach to calculate genetic contribution (i.e. LaGrange multipliers). However, small variations were observed between solutions of the two algorithms. These differences might be attributed to the fact that the Gencont algorithm obtains solutions by directly inverting the relationship matrix among selection candidates. However, Gencont2 uses Newton–Raphson steps to update Lagrangian multipliers and to solve for genetic contributions [Eq. (3)] with some degree of errors (10^−6^ convergence).

For the sheep dataset with 6895 selection candidates, Gencont could not be run because it could not handle such a large dataset due to the algorithm requiring repeated calculations of the inverse of the relationship matrices for the selection candidates [6]. However, the iterative algorithm was successful in obtaining optimized solutions and achieving predefined constraints. Table 4 presents the optimal number of rams to select and the genetic gain achieved at different rates of inbreeding for the sheep dataset. The optimal number of rams selected ranged from 25 to 107 when the constraint on the inbreeding rate became more stringent from 0.05 to 0.001 (Table 4).

**Table 4:** Analysis of the *Sheep dataset* using Gencont2 at different levels of rate of inbreeding ($\Delta F$) with respect to genetic gain ($\Delta G$), number of selected individuals and computer time

$\Delta F$	$\Delta G$	Ave_relationship^a^	Number of selected candidates	Time^b^
0.05	146.11	0.14922	25	8:27
0.01	140.03	0.07129	70	8:27
0.005	138.86	0.06156	89	8:12
0.001	137.70	0.05376	107	7:14

^a^Average relationship between selected candidates

^b^Amount of computation time necessary to find optimal solutions in minutes

These optimization analyses did not take any additional constraint on either the maximum or the minimum genetic contribution of a particular candidate into account. In practice, achieving optimal genetic contributions for all candidates may not be possible due to biological limitations and management policies. Such limitations can be incorporated in the optimization process by applying further restriction on minimal ($Cmin$) and maximal ($Cmax$) contributions (as shown in the Appendix). Table 5 summarizes the comparison of the two algorithms with the application of restrictions on minimal and maximal contributions for the cattle dataset. Table 5 presents the optimal number of candidates to select and $\Delta G$ under different combinations of $Cmax$ and $Cmin$ restrictions. If there was any difference between the two methods, then the results in parenthesis belong to Gencont. Table 5 shows that, for the cattle dataset, restriction on the maximal genetic contribution has more influence on the optimal number of candidates to select and genetic gain than the given restrictions on the minimal contribution.

**Table 5:** Analysis of the *Cattle dataset* with 3907 male selection candidates under different combinations of restrictions on the minimal and maximal contributions with respect to genetic gain and optimal number of candidates to select

$Cmin$	$Cmax$	$\Delta G$ ^a^	Number of selected animals^a^
0.0025	–	1.471	62 (65)
0.0050	–	1.471	51 (57)
–	0.01	1.437	100
–	0.02	1.464 (1.463)	76 (77)
–	0.03	1.470	74
–	0.04	1.472 (1.471)	75 (76)
–	0.05	1.472 (1.471)	75
0.0025	0.01	1.437	100
0.0025	0.02	1.462	68
0.0025	0.03	1.471 (1.470)	61 (62)
0.0025	0.04	1.471	64 (65)
0.0025	0.05	1.472 (1.471)	63 (65)
0.0050	0.01	1.437	100
0.0050	0.02	1.462	61
0.0050	0.03	1.470 (1.469)	53 (55)
0.0050	0.04	1.471	55 (56)
0.0050	0.05	1.471	57
0.0083	0.0083	1.429	120

$$Cmin$$ = minimum contribution

$$Cmax$$ = maximum contribution

$$\Delta F = 0.01$$

^a^If there was a difference between the two algorithms, the result obtained with Gencont is shown in parentheses

Table 6 presents the optimal number of candidates to select and $\Delta G$ under different combinations of $Cmax$ and $Cmin$ restrictions for the pig dataset. It also shows that restriction on the maximal genetic contribution has more effect on genetic gain than given restrictions on minimal contributions for the pig dataset. Scenarios where $Cmin = Cmax$ were also tested by fixing the number of individuals to select (Tables 5, 6).

**Table 6:** Analysis of the *Pig dataset* with 2929 selection candidates under different combinations of restrictions on the minimum and maximum contributions with respect to genetic gain and optimal number of candidates to select

$Cmin$	$Cmax$	$\Delta G$ ^a^	Number of selected animals^a^
0.0025	–	125.42 (125.39)	62 (68)
0.0050	–	125.34 (125.36)	48 (55)
–	0.01	123.77 (123.76)	102
–	0.02	124.97 (124.90)	84 (86)
–	0.03	125.19 (125.18)	81
–	0.04	125.34 (125.32)	78 (79)
–	0.05	125.37	77
0.0025	0.01	123.754	101
0.0025	0.02	125.05 (124.90)	76 (78)
0.0025	0.03	125.24 (125.18)	71 (73)
0.0025	0.04	125.33 (125.32)	70 (71)
0.0025	0.05	125.37	69
0.0050	0.01	124.97	100
0.0050	0.02	125.05 (124.90)	71
0.0050	0.03	125.24 (125.27)	66 (67)
0.0050	0.04	125.32	62
0.0050	0.05	125.54 (125.36)	56 (58)
0.0090	0.009	122.45 (122.36)	110

$$Cmin$$ = minimum contribution

$$Cmax$$ = maximum contribution

$$\Delta F = 0.01$$

^a^If there was a difference between the two algorithms, the result obtained with Gencont is shown in parentheses

Comparing results with and without restrictions on the maximal and minimal contributions (i.e. Table 2 vs. Table 5 and Table 3 vs. Table 6 for the cattle and pig datasets, respectively) shows that restriction on minimal contributions has very small or no effect on genetic gain. The main difference between these results is that candidates with the lowest contributions in the case of optimization without restriction are given zero contributions in the case of optimization with restriction on minimal contribution. However, restriction on maximal contribution has a notable effect on genetic gain and optimal number of individuals to select. These results are in agreement with Hinrichs et al. [11], who also reported that restriction on the minimal contribution has a limited effect on genetic gain compared with restriction on maximal contribution. However, the effect of restriction on minimal contribution could be significantly greater if a higher level of restriction was used.

Inbreeding is a growing concern in animal breeding programs. Advancements in statistical methods for genetic evaluation, such as BLUP, have increased the accuracy of estimated breeding values. This increase in accuracy comes with a cost of increasing the probability of co-selection of related individuals, which in turn increases the inbreeding level of a population (e.g. [1]). In the last decades, tools for genetic contribution optimization have been developed to manage rate of inbreeding in breeding programs [5, 6]. The use of OC selection has provided a useful tool to control the rate of at which inbreeding accumulates in a population. However, more importantly, OC selection has resulted in a higher genetic gain at the same level of inbreeding or in lower rate of inbreeding at the same level of genetic gain than traditional truncation selection [6].

One practical challenge in the use of the current existing OC selection algorithms, specifically Gencont, is the heavy computing requirements that arise from inverting the relationship matrix for large numbers of selection candidates repeatedly. To overcome this practical challenge, Hinrichs et al. [11] proposed an improvement to the Gencont algorithm. The main assumption in their method (OCSelect) is that finding the inverse of the relationship matrix between parents of the selection candidates is easier than between the candidates themselves because the size is relatively smaller. This might be true in some breeding programs; however, it might not be the case in dairy cattle and sheep breeding schemes, where the number of parents is large. Furthermore, the presence of overlapping generations (i.e. both parents and offspring could be selection candidates) in these species, may cause some computational problem in OCSelect. Methods such as that of Henderson [17] and Quaas [18] are available to derive directly the inverse of very large relationship matrices. These methods assume that the ${\mathbf{A}}^{{ - {\mathbf{1}}}}$ of all animals in the pedigree are required. However, in optimal contribution selection only the inverse of the relationship matrix between selection candidates is required. In addition, following the setup of Gencont, the ${\mathbf{A}}^{{ - {\mathbf{1}}}}$ had to be recalculated several times because candidates with invalid assigned contributions are rejected from the optimization process.

We present an alternative approach that uses an iterative algorithm and that can be applied to calculate optimal contributions. The iterative algorithm replaced the procedure that requires inversion of the relationship matrix repeatedly by obtaining solutions iteratively. In general, the results of the comparison between the two algorithms (i.e. Gencont2 and Gencont) showed that, in most of cases, both algorithms gave very similar solutions with respect to genetic gain and optimal number of candidates to select. In cases where the optimal number of candidates to select varied, differently selected candidates had very low contributions that ranged from 0.006 to 0.08 %. Furthermore, both algorithms constrained $\Delta F$ to the predefined levels. In agreement with previous studies [8, 10], the number of animals selected increased and genetic gain decreased as more severe constraints were placed on $\Delta F$. To satisfy the more stringent constraints, the degree of relationship among selection candidates needs to be reduced which is achieved by reducing the variance of ${\mathbf{c}}$. This means selecting more animals (assigning nonzero contributions to animals with lower EBV) and reducing the contribution of superior animals.

The setting up of constraints in Gencont can result in some individuals that have low breeding values being assigned invalid negative contributions. This problem is solved by fixing the contributions of these candidates to zero and repeating the optimization process without these candidates [6]. This process is repeated with fewer and fewer candidates until no negative contributions are found. Note that once a candidate is eliminated from the optimization process, it is no longer considered in the next iterations. This shows that Gencont does not guarantee that the solutions are always optimal and, under some circumstances, it might yield suboptimal solutions. Pong-Wong and Woolliams [7] worked out some numerical examples and demonstrated that Gencont does not guarantee that the final solutions are global maximum, but rather suboptimal under some scenarios. The same example is worked out here (see Appendix 2) to test Gencont2 in a similar situation. For the tested scenarios, Gencont2 found global maximum solutions that were similar to the solutions found by the semi-definite programming (SDP) method [7]. This is because Gencont2 does not rely on the elimination of candidates with assigned negative contributions, but rather implements some sort of constraint (i.e. $c_{i} \ge 0$) to make sure all solutions are valid.

There are other alternative algorithms for solving the optimization problem presented in this paper (i.e. objective function $H$) such as evolutionary algorithms [12], differential evolution [13] and semi-definite programming [7]. Evolutionary algorithms are more flexible and have the benefit of accommodating many practical constraints that would be challenging to deal with the Lagrangian multiplier approach. Semi-definite programming is well suited in optimization problems where the restrictions of the objective function are convex [7]. Differential evolution algorithms are a family of evolutionary algorithms and it has been shown that it is powerful to optimize diverse objective functions and is feasible for practical applications [13].

In step 1 of the iterative algorithm, it is assumed that ${\mathbf{A}}^{{ - {\mathbf{1}}}} = {\mathbf{I}}$ to calculate initial values for $\lambda_{0}$. At first look, this assumption seems very strong, however, it has limited or no impact on the final optimal solutions because in consecutive iterations the value of $\lambda_{0}$ is updated (i.e. in step 3) using *c* values obtained by solving ${\mathbf{Ac}} = \left( {{\mathbf{EBV}} - {\mathbf{Q}}{\varvec{\uplambda}}} \right)/2\lambda_{0}$. In addition, the iterative process will not converge before stabilizing the $\lambda_{0}$ value between consecutive iterations. The results also showed that the final solutions of the two algorithms are virtually similar with correlations being very close to 1 (Tables 2, 3). Thus, the iterative algorithm finds optimal contributions without inverting the relationship matrix ${\mathbf{A}}$.

In the case of genomic selection (GS), selection may concentrate on some chromosomal segments from generation to generation, because there are some genes with a larger effect on these segments [19]. It may be noted that the objective function $H$ could be extended to constrain several (genomic) relationship matrices, which may be relevant if inbreeding is to be constrained at several positions in the genome. This would require updating several $\lambda_{0}$ values in step 3 of the algorithm. As indicated by Sonesson et al. [20], when genomic selection is used, the pedigree relationship matrix ${\mathbf{A}}$ should be replaced by the marker-based (genomic) relationship matrix, ${\mathbf{G}}$, to constraint inbreeding in OC selection. The presented OC algorithm does not require the inverse of ${\mathbf{G}}$, which is often computationally difficult to obtain.

## Conclusions

The management of inbreeding in a breeding scheme requires that the average relationship between selected parents be managed. Optimal contribution selection is a useful tool to control rate of inbreeding while improving genetic gain. An iterative algorithm based on Meuwissen’s dynamic selection algorithm for calculating optimal contributions was developed. The presented iterative algorithm achieved a reduction in computer time of 90 to 93 % compared to the original algorithm and was able to handle datasets with a large number of selection candidates. The main advantage of the iterative algorithm is that it avoids (repeatedly) inversion of the relationship matrix. Thus, this iterative algorithm makes the implementation of optimal contribution selection for large-scale practical data possible, and thus enables the management of genetic diversity in breeding programs and increases their sustainability.

## Figures and Tables

**Fig. 2:**
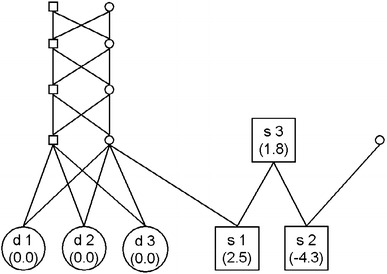
Pedigree structure for the example data. The values in brackets are estimated breeding values and the relationships were calculated based on the pedigree (from Pong-Wong and Woolliams [7])

## Appendix 1: Calculation of optimized genetic contributions with restriction on maximal and minimal contributions

The Appendix in Meuwissen [6] extended the dynamic optimal contribution algorithm for cases where some candidates have fixed contributions due to biological limitations and management policies. Here, the iterative algorithm is extended to handle such cases. The main purpose of this modification is to calculate the optimal contributions of the remaining candidates in the optimization process if the contributions of some candidates are set to fixed values. Let the remaining candidates in the optimization processes termed group 1 and candidates with fixed contribution termed group 2.

The objective function $H$ for this case is:

$$H = {\mathbf{c}}_{{\mathbf{1}}}^{T} {\mathbf{EBV}}_{{\mathbf{1}}} - \lambda_{0} \left( {{\mathbf{c}}_{{\mathbf{1}}}^{T} {\mathbf{A}}_{{{\mathbf{11}}}} {\mathbf{c}}_{{\mathbf{1}}} + 2{\mathbf{c}}_{{\mathbf{1}}}^{T} {\mathbf{A}}_{{{\mathbf{12}}}} {\mathbf{c}}_{{\mathbf{2}}} - K} \right) - {\varvec{\uplambda}}\left( {{\mathbf{c}}_{{\mathbf{1}}}^{T} {\mathbf{Q}}_{{\mathbf{1}}} - {\mathbf{s}}^{T} } \right),$$

where ${\mathbf{c}}_{{\mathbf{1}}}$ is a vector of the genetic contributions of group 1 candidates that are to be optimized, ${\mathbf{c}}_{{\mathbf{2}}}$ is a vector of known contributions of group 2 candidates, ${\mathbf{EBV}}_{{\mathbf{1}}}$ is a vector of breeding values of group 1 candidates, $K = 2\bar{C} - {\mathbf{c}}_{{\mathbf{2}}}^{T} {\mathbf{A}}_{{{\mathbf{22}}}} {\mathbf{c}}_{{\mathbf{2}}}$, $\bar{C}$ is the required average coefficient of co-ancestry for all candidates (i.e. $\bar{C} = C^{T} {\mathbf{A}}C^{T}$), ${\mathbf{A}}_{{{\mathbf{11}}}}$, ${\mathbf{A}}_{{{\mathbf{12}}}}$ and ${\mathbf{A}}_{{{\mathbf{22}}}}$ are part of the additive relationship matrix belonging to the candidates in group 1 and 2, ${\mathbf{s}} = {\mathbf{r}} - {\mathbf{Q}}_{{\mathbf{2}}}^{T} {\mathbf{c}}_{{\mathbf{2}}}$; ${\mathbf{r}}$ is a vector of 0.5 s of length two, ${\mathbf{Q}}_{{\mathbf{1}}}$ and ${\mathbf{Q}}_{{\mathbf{2}}}$ are known incidence matrices for the sex of candidates in group 1 and 2, respectively, and $\lambda_{0}$ and $\varvec{\uplambda}$ are Lagrangian multipliers.

Taking the gradient of $H$ with respect to ${\mathbf{c}}_{1}$ and equating to 0 yields:

5 $${\mathbf{c}}_{{\mathbf{1}}} = \frac{{{\mathbf{A}}_{{{\mathbf{11}}}}^{{ - {\mathbf{1}}}} \left( {{\mathbf{EBV}}_{{\mathbf{1}}} - 2\lambda_{0} {\mathbf{A}}_{{{\mathbf{12}}}} {\mathbf{c}}_{{\mathbf{2}}} - {\mathbf{Q}}_{{\mathbf{1}}} {\varvec{\uplambda}}} \right)}}{{2\lambda_{0} }},$$

From the constraint $K = 2\bar{C} - {\mathbf{c}}_{{\mathbf{2}}}^{T} {\mathbf{A}}_{{{\mathbf{22}}}} {\mathbf{c}}_{{\mathbf{2}}}$ follows an equation for $\lambda_{0}$:

6 $$\lambda_{0}^{2} = \frac{{\frac{1}{4} {\mathbf{EBV}}_{{\mathbf{1}}}^{T} {\mathbf{P}} {\mathbf{EBV}}_{{\mathbf{1}}} }}{{K + {\mathbf{c}}_{{\mathbf{2}}}^{{{T}}} {\mathbf{A}}_{{{\mathbf{21}}}} {\mathbf{PA}}_{{{\mathbf{12}}}} {\mathbf{c}}_{{\mathbf{2}}} - {\mathbf{s}}^{T} \left( {{\text{Q}}^{{T}} {\mathbf{A}}^{{ - {\mathbf{1}}}} {\mathbf{Q}}} \right)^{{ - {\mathbf{1}}}} {\mathbf{s}} - 2{\mathbf{s}}^{T} \left( {{\mathbf{Q}}^{{T}} {\mathbf{A}}^{{ - {\mathbf{1}}}} {\mathbf{Q}}} \right)^{ - 1} {\mathbf{Q}}_{{\mathbf{1}}}^{T} {\mathbf{A}}_{{{\mathbf{11}}}}^{{ - {\mathbf{1}}}} {\mathbf{A}}_{{{\mathbf{12}}}} {\mathbf{c}}_{{\mathbf{2}}} }}$$

where ${\mathbf{P}} = {\mathbf{A}}_{{{\mathbf{11}}}}^{{ - {\mathbf{1}}}} - {\mathbf{A}}_{{{\mathbf{11}}}}^{{ - {\mathbf{1}}}} {\mathbf{Q}}_{{\mathbf{1}}} \left( {{\mathbf{Q}}_{{\mathbf{1}}}^{T} {\mathbf{A}}_{{{\mathbf{11}}}}^{{ - {\mathbf{1}}}} {\mathbf{Q}}_{{\mathbf{1}}} } \right)^{ - 1} {\mathbf{Q}}_{{\mathbf{1}}}^{T} {\mathbf{A}}_{{{\mathbf{11}}}}^{{ - {\mathbf{1}}}}$. For a more detailed derivation of Eqs. (5) and (6) see Appendix of Meuwissen [6]. The procedure to calculate optimal contributions iteratively is described below.

Step 1: Calculate $\lambda_{0}$ by solving Eq. (6) (assuming ${\mathbf{A}}_{{{\mathbf{11}}}}^{{ - {\mathbf{1}}}} = {\mathbf{I}}$) and initiate some values for ${\varvec{\uplambda}}$ (for instance zero). Calculate optimal contributions for group 1 individuals, ${\mathbf{c}}_{1}$, by solving Eq. (5), i.e. ${\mathbf{A}}_{{{\mathbf{11}}}} {\mathbf{c}}_{{\mathbf{1}}} = \left( {{\mathbf{EBV}}_{{\mathbf{1}}} - 2\lambda_{0} {\mathbf{A}}_{{{\mathbf{12}}}} {\mathbf{c}}_{{\mathbf{2}}} - {\mathbf{Q}}_{{\mathbf{1}}} {\varvec{\uplambda}}} \right)/2\lambda_{0}$, using the Gauss–Seidel method.

Step 2: Update ${\varvec{\uplambda}}$ by taking the gradient of Eq. (5) with respect to ${\varvec{\uplambda}}$ and rearranging:

$${\varvec{\partial }}{\mathbf{c}}_{{\mathbf{1}}} /{\varvec{\partial }}{\varvec{\uplambda}} = - {\mathbf{A}}_{{{\mathbf{11}}}}^{{ - {\mathbf{1}}}} {\mathbf{Q}}_{{\mathbf{1}}} /2\lambda_{0} ,$$

$${\mathbf{Q}}_{{\mathbf{1}}}^{{T}} {\varvec{\partial}} {\mathbf{c}}_{{\mathbf{1}}} = - \frac{{{\mathbf{Q}}_{{\mathbf{1}}}^{T} {\mathbf{A}}_{{{\mathbf{11}}}}^{{ - {\mathbf{1}}}} {\mathbf{Q}}_{{\mathbf{1}}} \partial {\varvec{\uplambda}}}}{{2\lambda_{0} }},$$

We want to change ${\mathbf{c}}_{{\mathbf{1}}}$ by ${\varvec{\partial }}{\mathbf{c}}_{{\mathbf{1}}}$ such that $\Delta {\mathbf{s}} - {\mathbf{Q}}_{{\mathbf{1}}}^{{{T}}} {\varvec{\partial }}\varvec{c}_{{\mathbf{1}}} = 0$ (i.e. $\Delta {\mathbf{s}} = {\mathbf{Q}}_{{\mathbf{1}}}^{{{T}}} {\varvec{\partial }}{\mathbf{c}}_{{\mathbf{1}}}$). From the constraint ${\mathbf{Q}}_{{\mathbf{1}}}^{{{T}}} {\mathbf{c}}_{{\mathbf{1}}} = {\mathbf{s}}$, $\Delta {\mathbf{s}} = {\mathbf{s}} - {\mathbf{Q}}_{{\mathbf{1}}}^{{{T}}} {\mathbf{c}}_{{\mathbf{1}}}$ and assuming ${\mathbf{A}}_{{{\mathbf{11}}}}^{{ - {\mathbf{1}}}} = {\mathbf{I}}$:

7 $$\begin{aligned} & {\varvec{\partial }}{\varvec{\uplambda}} = - 2*\left( {{\mathbf{Q}}_{{\mathbf{1}}}^{{{T}}} {\mathbf{Q}}_{{\mathbf{1}}} } \right)^{ - 1} *\Delta {\mathbf{s}}*\lambda_{0} , \\ & {\varvec{\uplambda}}_{{{\mathbf{new}}}} = {\varvec{\uplambda}} + \partial \lambda \\ \end{aligned}$$

Step 3: Update $\lambda_{0}$. Equation (5) can be also written as:

8 $${\mathbf{A}}_{{{\mathbf{12}}}} {\mathbf{c}}_{{\mathbf{1}}} = \left[ {\frac{{\left( {{\mathbf{EBV}}_{{\mathbf{1}}} - {\mathbf{Q}}_{{\mathbf{1}}} {\varvec{\uplambda}}} \right)}}{{2\lambda_{0} }} - {\mathbf{A}}_{{{\mathbf{12}}}} {\mathbf{c}}_{{\mathbf{2}}} } \right],$$

Taking the gradient of Eq. (8) with respect to $\lambda_{0}$ and rearranging:

$${\varvec{\partial }}{\mathbf{c}}_{{\mathbf{1}}} = - \frac{{{\mathbf{A}}_{{{\mathbf{11}}}}^{{ - {\mathbf{1}}}} \left( {{\mathbf{EBV}}_{{\mathbf{1}}} - {\mathbf{Q}}_{{\mathbf{1}}} {\varvec{\uplambda}}} \right)\partial \lambda_{0} }}{{2\lambda_{0}^{2} }},$$

We want to change ${\mathbf{c}}_{{\mathbf{1}}}$ by ${\varvec{\partial }}{\mathbf{c}}_{{\mathbf{1}}}$ such that $\Delta K - {\mathbf{c}}_{{\mathbf{1}}}^{{{T}}} {\mathbf{A}}{\varvec{\partial }}{\mathbf{c}}_{{\mathbf{1}}} - 2{\varvec{\partial }}{\mathbf{c}}_{{\mathbf{1}}}^{{{T}}} {\mathbf{A}}_{{{\mathbf{12}}}} {\mathbf{c}}_{{\mathbf{2}}} = 0$ (i.e. that ${{\Delta }}K = {\mathbf{c}}_{{\mathbf{1}}}^{{{T}}} {\mathbf{A}}{\varvec{\partial }}{\mathbf{c}}_{{\mathbf{1}}} + 2{\varvec{\partial }}{\mathbf{c}}_{{\mathbf{1}}}^{{{T}}} {\mathbf{A}}_{{{\mathbf{12}}}} {\mathbf{c}}_{{\mathbf{2}}}$). Based on the constraint $K = {\mathbf{c}}_{{\mathbf{1}}}^{{{T}}} {\mathbf{A}}_{{{\mathbf{11}}}} {\mathbf{c}}_{{\mathbf{1}}} + 2{\mathbf{c}}_{{\mathbf{1}}}^{{{T}}} {\mathbf{A}}_{{{\mathbf{12}}}} {\mathbf{c}}_{{\mathbf{2}}}$, ${{\Delta }}K = K -{\mathbf{c}}_{{\mathbf{1}}}^{{{T}}} {\mathbf{A}}_{{{\mathbf{11}}}} {\mathbf{c}}_{{{\mathbf{11}}}} - 2{\mathbf{c}}_{{\mathbf{1}}}^{{{T}}} {\mathbf{A}}_{{{\mathbf{12}}}} {\mathbf{c}}_{{\mathbf{2}}}$. Taking gradient of ${{\Delta }}K$ with respect to ${\mathbf{c}}_{{\mathbf{1}}}$ and rearranging:

9 $$\partial \lambda_{0} = - \frac{{ {{\Delta }}K\lambda_{0}^{2} }}{{\left( {{\mathbf{c}}_{{\mathbf{1}}} + {\mathbf{A}}_{{{\mathbf{11}}}}^{{ - {\mathbf{1}}}} {\mathbf{A}}_{{{\mathbf{12}}}} {\mathbf{c}}_{{\mathbf{2}}} } \right)\left( {{\mathbf{EBV}}_{{\mathbf{1}}} - {\mathbf{Q}}_{{\mathbf{1}}} {\varvec{\uplambda}}} \right)}},$$

$$\lambda_{{0}_{new}} = \lambda_{0} + \partial \lambda_{0} ,$$

The term ${\mathbf{A}}_{{{\mathbf{11}}}}^{{ - {\mathbf{1}}}} {\mathbf{A}}_{{{\mathbf{12}}}} {\mathbf{c}}_{{\mathbf{2}}}$ in Eq. (9) is obtained iteratively.

Step 4: Re-calculate optimal contributions for the updated values for $\lambda_{{0_{new} }}$ and $\lambda_{new}$ by solving Eq. (5) for ${\mathbf{c}}_{{\mathbf{1}}}$, i.e. by solving ${\mathbf{A}}_{{{\mathbf{11}}}} {\mathbf{c}}_{{\mathbf{1}}} = \left( {{\mathbf{EBV}}_{{\mathbf{1}}} - 2\lambda_{0} {\mathbf{A}}_{{{\mathbf{12}}}} {\mathbf{c}}_{{\mathbf{2}}} - {\mathbf{Q}}_{{\mathbf{1}}} {\varvec{\uplambda}}} \right)/2\lambda_{0} .$

Step 5: Check for convergence and that the solutions are valid. The convergence was monitored after each round of iteration. If convergence is not achieved, then the algorithm will return back to step 2.

When solving Eq. (5) in steps 1 and 4 using the Gauss–Seidel method, solutions are constrained to be valid (i.e. $c_{i} \ge 0$). For computational reasons, animals with 0 contributions were removed after 500 iterations for the first time and after every 100th iteration until convergence.

## Appendix 2: A simple numerical example to demonstrate a scenario where Gencont gives suboptimal solutions and to compare results with Gencont2

Pong-Wong and Woolliams [7] demonstrated scenarios where suboptimal solutions are given by Gencont using simple numerical examples (Fig. 2). Here, we use the same example to demonstrate that the new algorithm (i.e. Gencont2) had improved the suboptimal solution problems in certain situations.

The example contains six candidates: three males and three females (Fig. 2). To simplify the illustration, only the contributions of the male candidates are optimized (female contributions are fixed to 1/6). The assumption is that similar behaviour may also be observed when optimizing a larger set of candidates. The authors calculated genetic contributions of the candidates using the semidefinite programming [7] method and Gencont method. Here, we compared the genetic contributions calculated by Gencont2 with the other two methods.

Breeding values of the candidates and pedigree information were from the example presented in Pong-Wong and Woolliams [7]. The example is designed to mimic a closed population in which external sires are introduced. The relationship between candidates was derived from the pedigree in Fig. 2. The methods were tested under the restriction of $K$ ≤ 0.29. The optimization process using Gencont2 assigned the contributions to 0.0495, 0.000, and 0.4505 for sires 1, 2, and 3, respectively, with an expected genetic gain of 0.935. This was the exact solution and expected genetic gain obtained by SDP [7]. They also tested all possible combinations and confirmed that this was the optimal solution. Gencont, on the other hand, assigned the contributions to 0.000, 0.000, 0.500 for sires 1, 2 and 3, respectively, with an expected genetic gain of 0.90 (3.7 % less than the gain obtained with SDP and Gencont2 methods). This is mainly because Gencont eliminates individuals with negative contributions at each iteration by assigning zero contributions. For this example, suboptimal solutions could be avoided if one candidate with the most negative solution is eliminated per iteration. However, in situations where the set of candidates is very large, the elimination of one candidate at a time may have some practical limitations.

Testing of the programs in other scenarios where there is restriction on the maximum and minimum contributions per individual, Gencont failed to find optimal solutions for some cases (see [7]). However, Gencont2 provided solutions similar to the SDP solutions for these cases. This can be attributed to the fact that Gencont2 does not remove animals with invalid solutions from the optimization process after each iteration but rather uses the Gauss–Seidel step to constrain the solutions to be valid.
